# Accelerated Curing and Enhanced Material Properties of Conductive Polymer Nanocomposites by Joule Heating

**DOI:** 10.3390/ma11091775

**Published:** 2018-09-19

**Authors:** Sung-Hwan Jang, Donghak Kim, Yong-Lae Park

**Affiliations:** 1 Civil and Coastal Engineering, School of Engineering, University of Plymouth, Plymouth, Devon PL4 8AA, UK; sung-hwan.jang@plymouth.ac.uk; 2 Department of Mechanical and Aerospace Engineering, Seoul National University, Seoul 08826, Korea; maxkim322@snu.ac.kr; 3 Soft Robotics Research Center (SRRC), Seoul National University, Seoul 08826, Korea; 4 Institute of Advanced Machines and Design (IAMD), Seoul National University, Seoul 08826, Korea

**Keywords:** particulate composites, carbon nanotubes, Joule heating, conductive polymers

## Abstract

Joule heating is useful for fast and reliable manufacturing of conductive composite materials. In this study, we investigated the influence of Joule heating on curing conditions and material properties of polymer-based conductive composite materials consisting of carbon nanotubes (CNTs) and polydimethylsiloxane (PDMS). We applied different voltages to the CNT nanocomposites to investigate their electrical stabilization, curing temperature, and curing time. The result showed that highly conductive CNT/PDMS composites were successfully cured by Joule heating with uniform and fast heat distribution. For a 7.0 wt % CNT/PDMS composite, a high curing temperature of around 100 °C was achieved at 20 V with rapid temperature increase. The conductive nanocomposite cured by Joule heating also revealed an enhancement in mechanical properties without changing the electrical conductivities. Therefore, CNT/PDMS composites cured by Joule heating are useful for expediting the manufacturing process for particulate conductive composites in the field of flexible and large-area sensors and electronics, where fast and uniform curing is critical to their performance.

## 1. Introduction

Attention has been paid to particulate polymer-based composites owing to their multifunctional characteristics, such as mechanical, electrical, and thermal properties [1,2,3,4,5,6,7,8,9]. In particular, conductive composites are generally obtained from networks of conductive fillers, such as metallic or carbon-based particles that increase the electrical conductivities of the composites [10]. Amongst many filler candidates, carbon nanotubes (CNTs) have widely been employed to make composites conductive due to their low density, non-toxicity, high electrical conductivity, and high aspect ratio [11,12,13].

In previous engineering applications, conventional oven heating was mostly used to cure polymer-based composites, but it was often observed that the heat distribution was not uniform, and that a relatively long period of heating was required during manufacturing. Therefore, internal heating methods, such as Joule, microwave, and induction heating, have been of great interest due to their non-contact heating process, which creates uniform thermal distribution and requires only short heating times [14,15,16,17,18]. In particular, Joule heating is one of the most effective ways to provide uniform heating of conductive composites. For carbon nanotube-reinforced composites, an electric field in the presence of an electric current does mechanical work by moving the current-carrying particles in the conductor [19,20,21]. This work can be dissipated into the heat in the conductor. Therefore, curing by Joule heating will be beneficial to various applications in the field of flexible and large-area sensors and electronics, where fast and uniform curing of the base polymer is critical to their performance.

In this study, particulate polymer-based conductive composites cured by Joule heating were characterized. The CNT nanocomposites were prepared by a simple solution-casting method for high and stable electrical conductivity. The nanocomposites were cured at different voltages to determine a curing temperature for fast curing and enhanced material properties. We observed the electrical stabilization and temperature profile of the CNT nanocomposites during the curing process. Finally, we assessed the mechanical and electrical properties of the CNT nanocomposites cured by Joule heating and compared them to those of samples cured by conventional oven heating.

## 2. Materials and Fabrication Methods

Multi-walled carbon nanotubes with an average length of 15 μm and average diameter of 10 nm were obtained from Nanolab (Waltham, MA, USA). Polydimethylsiloxane (PDMS) (Sylgard 184) was purchased from Dow Corning (Midland, MI, USA). The densities of the CNTs and the PDMS were 0.96 g/cm^3^ and 1.12 g/cm^3^, respectively.

**Figure 1:**
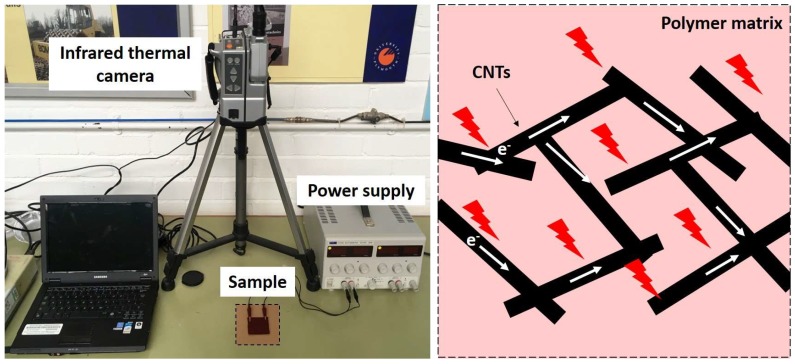
Experimental set-up for the fabrication of carbon nanotube (CNT) nanocomposites cured by Joule heating.

**Figure 2:**
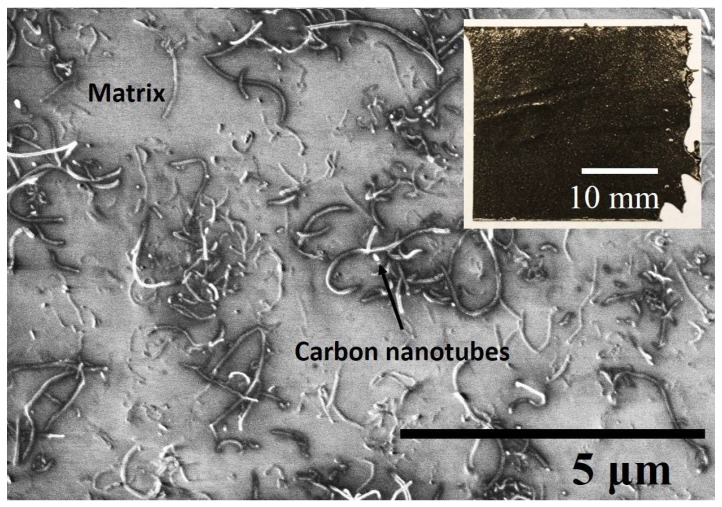
Scanning electron microscope (SEM) image of CNT distribution in the matrix.

Different concentrations of as-received CNTs were mixed with PDMS using a shear mixer (ARE301, Thinky Corp., Tokyo, Japan). It operated at 2200 rpm for three minutes at room temperature. A curing agent was added to the mixture at a weight ratio of 10:1 for the elastomer and the curing agent and mixed by the shear mixer for another three minutes. Then, the mixture was degassed in a vacuum chamber to eliminate air bubbles, which may cause lower electrical conductivity and poorer mechanical properties than desired, and poured into a mold. In order to cure the mixture by Joule heating, copper-based electrodes were attached to the ends of the mold. The curing temperature was controlled by adjusting the input voltage with a direct current (DC) power supply, as shown in Figure 1. The cured CNT nanocomposite was in the form of a flexible thin film, as shown in Figure 2. The dispersion of CNTs was randomly distributed in the matrix, forming CNT networks that resulted in the conductive composites. The measured conductivity of the fabricated composites was approximately 3.0 S/m.

A scanning electron microscope (FEI Sirion 600, JEOL, Tokyo, Japan) was used to observe the CNT dispersion in the matrix. The electrical resistances of the CNT nanocomposites were measured by digital multimeters. The samples were prepared in a square shape of 30 × 30 × 2 mm^3^. Then, the electrical conductivity *σ* of the samples was calculated by *σ* = *l*/(*A*·*R*), where *l*, *A*, and *R* are the length, the area, and the resistance of the sample, respectively.

The Joule heating was applied to the CNT nanocomposite (30 × 30 × 2 mm^3^) samples in a pre-polymer state by inducing different voltages using a DC power supply (6211A, Hewlett Packard, Palo Alto, CA, USA). Temperature was recorded using a digital thermocouple logger (SL500TC, Supco, Allenwood, NJ, USA), which was attached to the middle of the mold. Thermal images of the samples were also taken using a thermal infrared camera (TH7102WX, NEC, Tokyo, Japan). All tests related to Joule heating were conducted with samples with the dimensions of 30 mm × 30 mm × 2 mm.

The mechanical properties of the CNT nanocomposites cured by conventional oven heating and Joule heating were characterized using a motorized materials tester (ESM301L, Mark-10, Copiague, NY, USA). The samples were prepared in a rectangular shape 10 mm wide, 50 mm long, and 2 mm thick. A tensile test was performed at a rate of 30 mm/min at room temperature. A set of five specimens was used for each material to evaluate its electrical, thermal, and mechanical properties.

To evaluate the curing performances of the proposed Joule heating and the conventional oven heating methods, thermoanalytical experiments were conducted using a differential scanning calorimeter (DSC, Q100, Texas Instruments, Dallas, TX, USA). Two identical CNT nanocomposite samples with the same conductivity of approximately 3.0 S/m were prepared. One was cured by oven heating and the other was cured by Joule heating for the same period of time. The curing temperature of the Joule-heated sample was equivalent to that of the oven-heated one in this experiment. Each sample was placed in the DSC and heated at 70 °C for three minutes to measure the heat flow.

## 3. Results

### 3.1. Electrical Conductivity of CNT Nanocomposites

**Figure 3:**
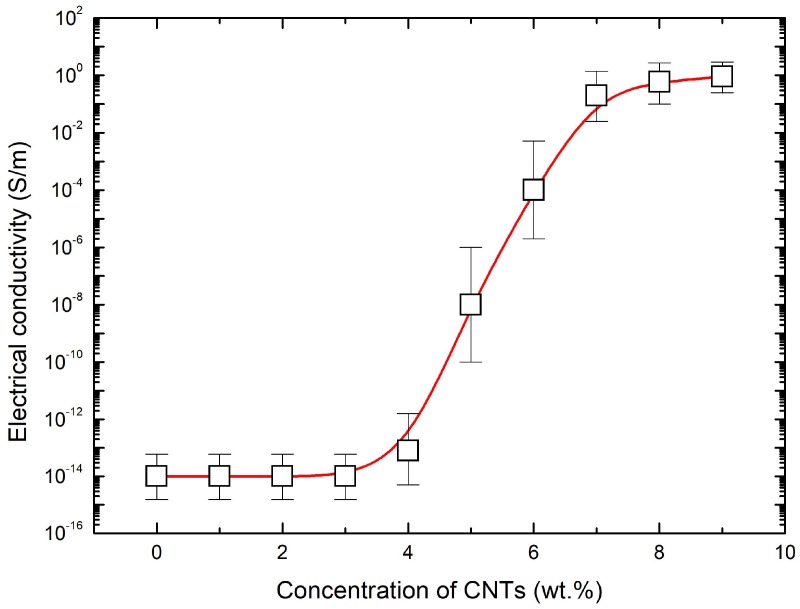
Electrical conductivity of CNT nanocomposites as a function of CNT concentration.

Figure 3 shows the electrical conductivity of the CNT nanocomposites as a function of CNT concentration. It is clear that the conductivity of the CNT nanocomposites significantly increased with an increase of the CNT concentration. At a low CNT concentration, CNTs did not form a continuous network in the matrix, resulting in a low electrical conductivity. Above a certain concentration, CNTs initially made a continuous network that became the path of electrons, which is called a percolation threshold. The electrical conductivity of the CNT composites was fitted to the relations of the percolation threshold law, as shown in Equation (1).

(1) $$\sigma_{DC}∼\{\begin{array}{l} {\left(\phi_{c}-\phi \right)}^{-s}\;\mathrm{when}\;\phi_{c}<\phi \\ {\left(\phi -\phi_{c}\right)}^{t}\;\;\mathrm{when}\;\phi <\phi_{c} \end{array}$$

where $\phi_{c}$ is the threshold volume fraction for electrical percolation, and *s* (~0.7) and *t* (~5.2) are critical exponents before and after the percolation threshold, respectively. In this study, the percolation threshold occurred at around 4.0 wt % of CNTs, which is a relatively higher value compared to the result of previous work, due to different fabrication methods [10,22,23,24]. At a high concentration of CNTs, the electrical conductivity gradually increased as a result of the full capacity of electron paths. For Joule heating applications controlled by input voltages, high electrical conductivity is useful in terms of energy efficiency. Therefore, for all further investigations, we used samples of CNT nanocomposites with 7.0 wt % of CNTs, whose electrical conductivity was approximately 3.0 S/m.

### 3.2. Current Stabilization

**Figure 4:**
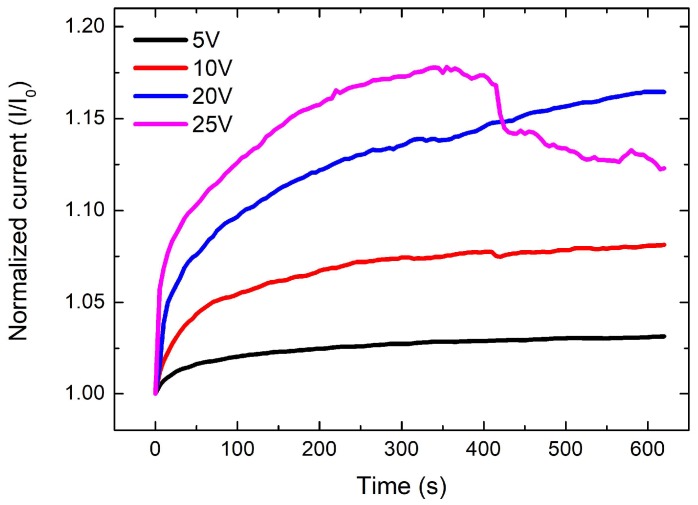
Change in normalized electric current of CNT nanocomposites as a function of curing time and applied voltages.

To demonstrate the electrical stabilization of CNT nanocomposites, we prepared CNT nanocomposite samples with 7.0 wt % of CNTs showing ~500 Ω at room temperature. Different DC voltages were then applied to the electrodes of the samples. Figure 4 shows the change in normalized electric current (*I*/*I*_0_) of nanocomposites with different voltages applied. An increase in current (or decrease in resistance), based on Ohm’s law, of all the samples was observed as time increased. This is because the CNT-polymer composites have a negative temperature coefficient (NTC) of resistance. An NTC occurs in materials that show a decrease in resistance when their temperature is increased due to the material properties, such as the thermal expansion coefficient, melting point, glass transition, and physical properties of fillers [25,26]. In particular, the NTC for CNT/PDMS composites was caused by the reduction in interlayer thickness between CNTs due to the thermal expansion of the PDMS during the heating [27]. All samples showed stable current changes except for the sample to which 25 V was applied. In this study, the nanocomposite under 25 V showed a sudden drop in current (i.e., a dramatic increase in resistance) during Joule heating, indicating a destruction of the CNT networks in the matrix due to the high concentration on voltage amongst CNTs [28]. Therefore, optimization of voltage was required for the fast curing application using Joule heating.

### 3.3. Fast Curing by Joule Heating

**Figure 5:**
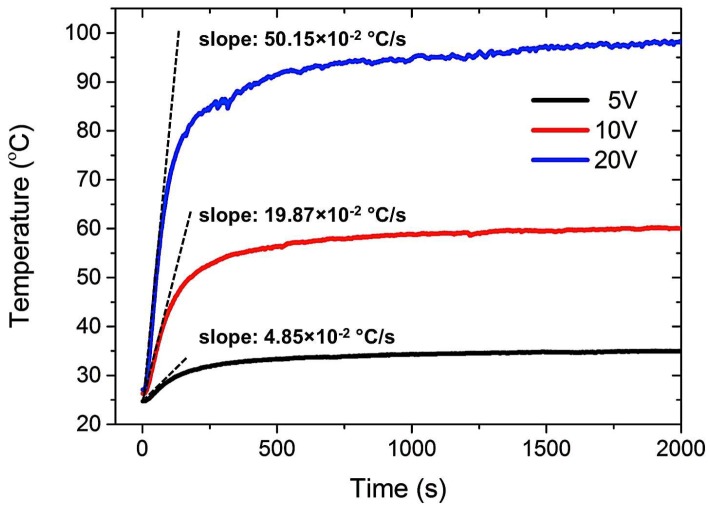
Temperature profiles of CNT nanocomposites cured at different voltages showing rapid temperature increase during the initial linear region and steady temperature states in the long term.

**Figure 6:**
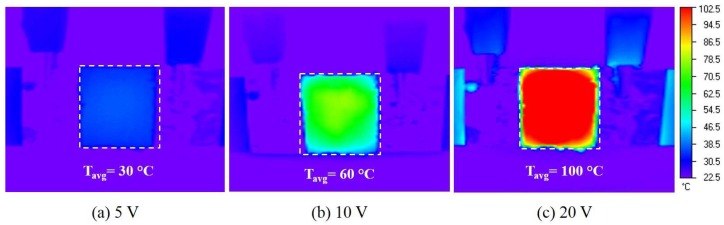
Infrared images showing temperature distributions of CNT nanocomposites cured at different voltages: (**a**) 5 V; (**b**) 10 V; (**c**) 20 V.

**Figure 7:**
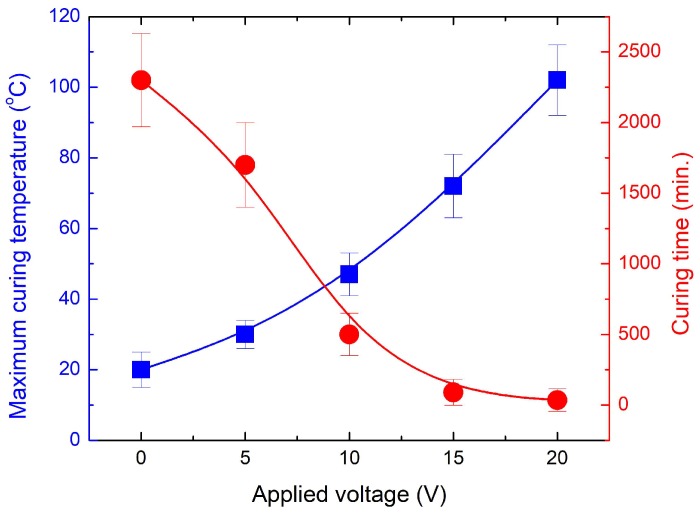
Maximum curing temperature and curing time for CNT nanocomposite cured at different applied voltages.

To investigate the effect of Joule heating on the curing temperature and time of CNT nanocomposites, different voltages (5, 10, 15, and 20 V) were applied to the pre-polymer state composites except for 25 V due to the instability observed in the previous section. Figure 5 shows the temperature profiles of the CNT nanocomposite cured at different voltages. The curing temperature of the nanocomposite dramatically increased with an increase of the applied voltage. In particular, the nanocomposite cured at a high voltage showed a significantly higher temperature increase rate in the initial linear region (approximately 0–100 s), as summarized in Table 1. For example, the initial temperature increase rate and the maximum temperature of the nanocomposite cured at 20 V are approximately 10 times and three times higher than those of the nanocomposite cured at 5 V and 10 V, respectively. In addition, after the initial temperature increase, the nanocomposite showed temperature stability over an extended period of time with all three voltages. Figure 6 presents the infrared images of the nanocomposites cured at different voltages with temperature distributions. The nanocomposites cured by Joule heating also showed uniform temperature distribution. Therefore, the elevated curing temperature of PDMS significantly reduced the curing time due to the higher degree of crosslinking of the PDMS network, as discussed in Reference [29]. Figure 7 shows the maximum temperature of the CNT nanocomposites and the curing time as a function of the applied voltages. The maximum curing temperature significantly increased with increasing voltages. For example, while the curing time of the nanocomposite at room temperature was around 24 h, the curing time of the nanocomposite to which 20 V was applied was only several minutes. The curing times of the Joule-heated samples and the oven-heated samples were the same. The polymerization of CNT nanocomposites cured by Joule heating and oven heating will be discussed in Section 3.4.

### 3.4. Effect of Joule Heating on Electrical and Mechanical Properties of CNT Nanocomposites

**Figure 8:**
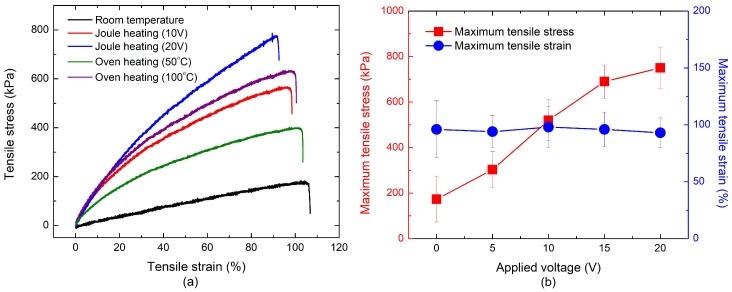
Mechanical properties of CNT nanocomposites cured by oven heating and Joule heating: (**a**) stress-strain curve for direct tensile testing of CNT nanocomposites; (**b**) maximum tensile strength and strain of CNT nanocomposites cured by Joule heating.

Representative tensile stress-strain curves for CNT nanocomposites cured by oven heating and Joule heating are shown in Figure 8a. Young’s modulus of the CNT nanocomposites was determined based on the secant modulus at the tensile strain of 50.0% considering the nonlinear behavior of the polymer-based composite. The nanocomposites cured by Joule heating showed higher stiffness than those cured by oven heating due to the uniform heat distribution of Joule heat. The nanocomposites cured at a higher voltage showed a higher stiffness compared with those cured at room temperature or at lower voltages. This indicates that the high internal curing temperature accelerated the cross-linking reaction rate of the polymer and increased the extent of cross-linking. Figure 8b shows a comparison of the maximum tensile stress and strain of CNT nanocomposites cured by Joule heating. The tensile strength of the samples cured by Joule heating increased as the applied voltages increased. For example, the maximum tensile strength of the samples cured at 10 V and 20 V was respectively three and four times higher than that of the samples cured at room temperature. However, a similar maximum tensile strain of the nanocomposites cured at different voltages was observed in this study.

**Figure 9:**
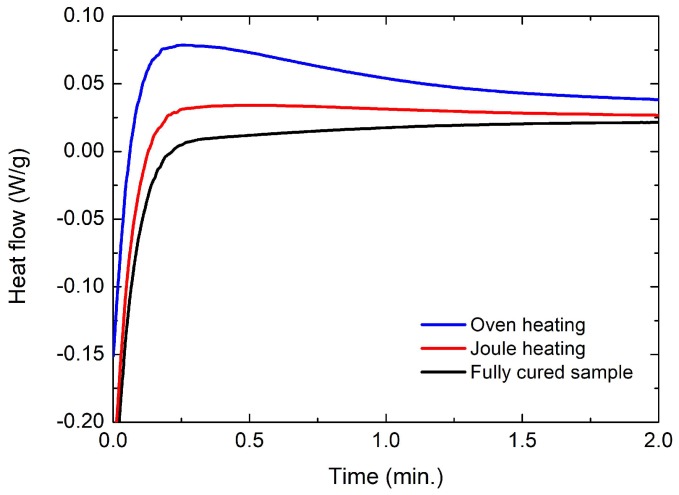
Isothermal differential scanning calorimeter (DSC) curves of CNT nanocomposites cured by Joule heating and oven heating.

The effect of curing by Joule heating can also be explained by thermoanalytical experiments conducted using a DSC. Figure 9 shows the heat flow of the CNT nanocomposite samples (~3.0 S/m) cured by oven heating and Joule heating. A much higher exothermic peak was observed in the sample cured by oven heating than in the sample cured by Joule heating, indicating higher and longer crosslinking reactions in the oven-heated polymer sample. This is because internal heating (i.e., Joule heating) creates a more uniform thermal area and rapid temperature increase than heating by convection (i.e., oven heating) [30]. Therefore, we can confirm that Joule heating expedites the curing process of CNT nanocomposites compared with conventional oven heating, leading to higher stiffness as well.

**Figure 10:**
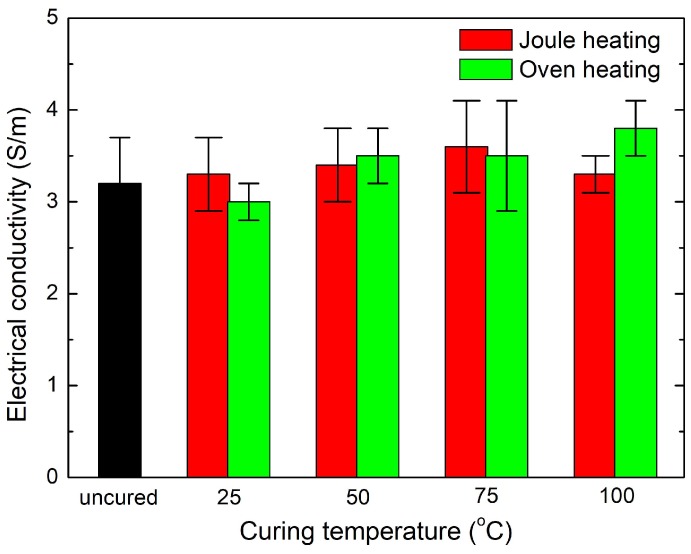
Electrical conductivity of CNT nanocomposites at different curing temperatures, comparing Joule heating and conventional oven heating.

Figure 10 demonstrates comparison of the electrical conductivities of the CNT nanocomposites cured at different temperatures through Joule heating and conventional oven heating. It is clearly observed that the electrical conductivity of the nanocomposites did not change regardless of different applied voltages and curing methods. This indicates that both curing methods did not affect the change in the CNT network since electrons only pass through the CNT networks in the composite due to the non-conductive polymer. Therefore, curing assisted by Joule heating is a useful manufacturing technology for conductive polymer-based composites that enhances production quality.

## 4. Conclusions

In summary, the effect of Joule heating on the curing time and the material characteristics of CNT nanocomposites was investigated in terms of both mechanical and electrical properties. Conductive polymer mixtures were prepared by mixing PDMS with multi-walled carbon nanotubes. The electrical conductivity of the CNT nanocomposites significantly increased with an increase of CNT concentrations. Then, highly conductive pre-polymer was cured at different voltages using a DC power supply. It was observed that too high an input voltage might impair the CNT networks during the curing process, which requires the selection of an appropriate operating condition when Joule heating is used for the curing of the CNT nanocomposite. In addition, our experimental results confirmed that curing by Joule heating (1) significantly reduced the curing time and (2) enhanced the mechanical properties of the CNT nanocomposites. However, the electrical conductivity of the composites cured by Joule heating was no different from that of the samples cured by conventional oven heating. Therefore, Joule heating not only expedites the manufacturing process, but also enhances the mechanical properties of conductive nanocomposites without degrading their electrical conductivity. One area of future work will be the investigation into the effect of Joule heating on the exothermic behavior of the induced polymerization in order to directly observe the Joule effect.

## Figures and Tables

**Table 1:** Temperature increase rate in the initial linear regions of CNT nanocomposites cured at different voltages.

**Applied Voltage**	5 V	10 V	20 V
**Temperature Increase Rate (×10^−2^ °C/s)**	4.85	19.87	50.15
